# Sclerotherapy in the treatment of varicose veins: S2k guideline of the Deutsche Gesellschaft für Phlebologie (DGP) in cooperation with the following societies: DDG, DGA, DGG, BVP

**DOI:** 10.1007/s00105-020-04705-0

**Published:** 2020-11-30

**Authors:** E. Rabe, F. X. Breu, I. Flessenkämper, H. Gerlach, S. Guggenbichler, B. Kahle, R. Murena, S. Reich-Schupke, T. Schwarz, M. Stücker, E. Valesky, S. Werth, F. Pannier

**Affiliations:** 1 Emeritus Klinik und Poliklinik für Dermatologie und Allergologie, Universitätsklinikum Bonn (AöR), Venusberg-Campus 1, 53127 Bonn, Germany; 2 Bad Wiessee, Germany; 3 Berlin, Germany; 4 Viernheim, Germany; 5 Praxis fürAllgemeinmedizin und Phlebologie, Munich, Germany; 6 Klinik für Dermatologie, Allergologie und Venerologie, Universitätsklinikum Schleswig-Holstein, Lübeck, Germany; 7 Phlebologische Praxis, Köln, Germany; 8 Privatpraxis für Haut- und Gefäßmedizin, Wundtherapie, Recklinghausen, Germany; 9 Praxis für Gefäßmedizin, Freiburg, Germany; 10 Klinik für Dermatologie, Venerologie und Allergologie, Ruhr-Universität Bochum, St. Josef Hospital, Bochum, Germany; 11 Klinik für Dermatologie, Venerologie und Allergologie, Universitätsklinik Frankfurt, Frankfurt, Germany; 12 Universitäts-Gefäßzentrum, Innere Medizin III, Universitätsklinikum Carl Gustav Carus, Dresden, Germany; 13 Praxis Dermatologie & Phlebologie Bonn und Dermatologische Universitätsklinik Köln, Bonn, Germany

These guidelines are also consistent with the results of the first European guidelines for sclerotherapy dating from 2012 [1]. These guidelines consider the current state of the literature, but not in every case the different conditions of approval of the various drugs.

## 1 Definition

Sclerotherapy is the targeted chemical ablation of a varicose vein by intravenous injection of a liquid or foam sclerosant [1, 2]. Intradermal, subcutaneous and/or transfascial (perforator) veins can be treated by this method, as well as epi-, supra- and subfascial vessels with venous malformations. The sclerosant destroys the endothelium of the vein and possibly other regions of the vein wall, and is deactivated by blood components and circulating cells [1, 2]. After successful sclerotherapy the varicose vein is transformed in the long term into a string of connecting tissue, in a process known as sclerosis [3–6]. The objective of sclerotherapy is not thrombosis of the vein, since re-channelling may occur after this process, but its transformation into a string of connecting tissue. Re-channelling of this is impossible, so the functional outcome is equivalent to removal of the vein or endovenous thermal ablation.

## 2 Objectives of sclerotherapy

The objectives of sclerotherapy are:

- Ablation of varicose veins.
- Prevention and treatment of complications of chronic vein disease.
- Improvement and/or elimination of venous symptoms, improved quality of life.
- Improved venous function.
- Improved aesthetic appearance.

The objectives are consistent with those of other therapeutic procedures for varicose veins.

## 3 Indications

### Recommendation 1

Sclerotherapy can be used for all forms of varicose veins, especially:

- Incompetent saphenous veins [5, 7–18].
- Varicose tributaries [19, 20].
- Incompetent perforator veins [19, 21–24].
- Reticular varices [8, 20, 25–30].
- Spider veins (telangiectasia) [8, 20, 25–30].
- New, remaining and recurrent varicose veins after previous operations [19, 31–39].
- Genital and perigenital varices [31, 40–42].
- Varicose veins (veins with reflux) around a leg ulcer [43–50].
- Venous malformations [51–57].

Other indications (e.g. varicose veins in the oesophagus, haemorrhoids, varicocele, hygroma, lymphatic cyst, Baker’s cyst) are not covered by these guidelines.

Treatment with liquid sclerosants is considered the method of choice for reticular varices and spider veins due to the stability of the available data (C1 varicose veins according to the CEAP classification) [25, 27, 29, 58, 59]. Foam sclerotherapy is an additional treatment option for C1 varicose veins [8, 28, 29, 60].

Thermal and operative procedures have been established for the treatment of varicose saphenous veins. The treatment of incompetent saphenous veins by sclerotherapy is likewise a successful and cost-efficient treatment option [18, 61–70]. It has comparatively few side effects and can be repeated as required. This is particularly true of foam sclerotherapy, as has been shown in recent years by case studies and prospective, randomised, controlled trials [5, 11, 18, 27, 65, 66, 71–73]. The re-channelling and recurrence rates are higher than with operative and thermal procedures [11, 14–17]; however, the improvement in quality of life achieved after 5 years is similar to that of EVLA and stripping operations [18].

In combination with other saphenous vein ablation procedures, sclerotherapy with percutaneous vein ablation is an option for the elimination of an accompanying varicose tributary, either in the same session or after an interval [19, 20]. The same is true of treatment of recurrent varicose veins [37, 38]. Early ablation of the incompetent saphenous vein as well as peri-ulcer sclerotherapy has proved effective in the treatment of venous leg ulcers. Foam sclerotherapy of the incompetent saphenous vein accelerates ulcer healing, comparable with endovenous thermal procedures [74].

## 4 Contraindications

### Recommendation 2

The following absolute and relative contraindications for sclerotherapy shall be observed:

Absolute contraindications [3, 4, 7, 59, 75]:

- Known allergy to the sclerosant.
- Acute venous thromboembolism.
- Local infection in the region of the sclerotherapy or severe generalised infection.
- For foam sclerotherapy:
  Known symptomatic right-to-left shunt (e.g. symptomatic patent foramen ovale).

Relative contraindications (individual risk–benefit assessment is obligatory) [3, 59, 76]:

- Pregnancy.
- Lactation (if the indication is urgent, interrupt lactation for 2–3 days).
- Severe peripheral arterial occlusive disease.
- Poor general state of health.
- High risk of thromboembolism (e.g. known history of thromboembolic events, known severe thrombophilia, active cancer).
- Long-term immobility or bed-ridden patient.
- For foam sclerotherapy:
  Neurological disorders, including migraine, after previous foam sclerotherapy.

Anticoagulation treatment is not a contraindication for sclerotherapy per se [43, 77, 78]; however, patients should be advised that the success of the treatment may be reduced and/or several treatments may be needed.

In addition, the technical information current in Germany, the instruction leaflet or the product description for the sclerosant used should be observed.

## 5 Complications and risks

If correctly executed, sclerotherapy is an efficient form of treatment with few complications [79].

### Recommendation 3

Care shall be taken with the following undesired events after sclerotherapy [80–86] (GRADE IB) (Table 1):

**Table 1:** Undesired events after sclerotherapy. (Modified and updated from [81])

**Description**	**Frequency**	
***** Very frequent	≥10%	
**** Frequent	≥1–<10%	
*** Occasional	≥0.1–<1%	
** Rare	≥0.01–<0.1%	
* Very rare and individual cases	<0.01%	
**Type of undesired event**	**Frequency**	
	**With liquid sclerotherapy**	**With foam sclerotherapy**
*Serious complications* ^*a*^		
Anaphylaxis	* Individual cases	* Individual cases
Extensive tissue necrosis	* Individual cases	* Individual cases
Stroke and TIA	* Individual cases	* Individual cases
Distal deep vein thrombosis (usually muscular)	** Rare	*** Occasional
Proximal deep vein thrombosis	* Very rare	* Very rare
Lung embolism	* Individual cases	* Individual cases
Damage to motor nerves	* Individual cases	* Individual cases
*Benign complications*		
Visual disturbances	* Very rare	*** Occasional
Headache and migraine	* Very rare	*** Occasional
Damage to sensory nerves	* Not reported	** Rare
Tightness in the chest	* Very rare	* Very rare
Dry cough	* Very rare	* Very rare
Superficial thrombosis	Unclear^b^	Unclear^b^
Skin reaction^c^ (contact allergy)	* Very rare	* Very rare
Matting	**** Frequent	**** Frequent
Hyperpigmentation	**** Frequent	**** Frequent
Skin necrosis (minimal)	** Rare	* Very rare
Embolia cutis medicamentosa	* Very rare	* Very rare

^a^As with all medication treatments, the possibility cannot be excluded that some of these serious side effects (e.g. anaphylaxis) may be fatal in the worst cases

^b^Frequencies between 0 and 45.8% are reported in the literature, with a mean value of 4.7% (see below)

^c^Local wheal formation and urticaria factitia may be observed at the insertion point, similar to that observed in the context of local histamine release; these should not generally be considered an “allergic reaction”

### Anaphylaxis

Anaphylactic shock is an extremely rare complication, which shall be treated as an emergency [87, 88].

#### Recommendation 4

If an anaphylactic reaction is suspected, injection shall be stopped immediately and the usual emergency measures taken, including, if appropriate, administering anti-histamines, corticosteroids and epinephrine.

### Extensive tissue necrosis

Extensive necrosis may appear after inadvertent intra-arterial injection [89–92]. The risk of intra-arterial injection can be minimised by ultrasound control with proper representation and identification of the arteries in the immediate neighbourhood of the target veins. If severe pain occurs during injection, the procedure should be stopped immediately. If intra-arterial injection is suspected, local anticoagulation and thrombolysis should be administered by catheter if possible. This can be complemented, if appropriate, by systemic anticoagulation. Prompt administration of systemic corticosteroids can help to reduce the inflammatory reaction [85, 89].

#### Recommendation 5

To avoid inadvertent perivenous or intra-arterial injection, in both foam and liquid sclerotherapy, the injection should be carried out under ultrasound control if the vein cannot be seen or felt easily and safely.

#### Recommendation 6

If intra-arterial injection is suspected, local anticoagulation and thrombolysis should be administered by catheter if possible; this can be complemented, if appropriate, by systemic anticoagulation. Prompt administration of systemic corticosteroids can help to reduce the inflammatory reaction.

### Skin necrosis and embolia cutis medicamentosa

Skin necrosis is described both after perivascular injection of high-percentage sclerosant and in rare cases after correct intravascular injection of the sclerosant at low concentrations [93]. However, it has been shown that subcutaneous perivascular injection of liquid or foam polidocanol is not responsible for skin necrosis after sclerotherapy of reticular varices or spider veins [94]. In these cases a mechanism is assumed with transfer of sclerosant into a leg artery through an arteriovenous anastomosis or venoarterial reflex vasospasm [85, 95, 96]. In individual cases this has been described as embolia cutis medicamentosa or Nicolau syndrome [97, 98]. Treatment of skin necrosis should follow the recommendations for general wound treatment. Healing can sometimes be protracted.

#### Recommendation 7

To reduce the risk of skin necrosis, injection of large volumes at any injection point should be avoided. The sclerosant should be injected at the lowest possible pressure.

### Vision disorders, headache and migraine

Transient migraine-like symptoms can be observed after all forms of sclerotherapy. They appear more frequently after foam sclerotherapy than liquid sclerotherapy [58, 80, 84, 99–102]. To date no pathological findings have been reported in ophthalmological research, and there are no reports of lasting vision disorders [100].

Right-to-left shunt, for example due to patent foramen ovale, occurs in around 30% of the population; discussion continues as to whether the transfer of foam bubbles into the arterial circulation plays a part in this condition [103–107].

Vision disorders after sclerotherapy probably reflect a migraine with aura rather than temporary ischaemic cerebrovascular events [108, 109].

Vision disorders may be accompanied by paraesthesia and dysphasic speech disorders, depending on the extent of cortical spreading depression, the pathological equivalent of migraine with aura. There is no firm proof of interdependence between foam bubbles and visual or neurological disorders. Recent data show that potentially vasospastic endothelin 1 is released from vessels into which liquid or foam sclerosant has been injected [110, 111]. Vision disorders occur in patients with a history of migraine more frequently than in patients with no such history [108]. Multiple injections of small doses may possibly reduce rapid transfer of the sclerosant into the deep veins [112].

### Stroke and transient ischemic attack (TIA)

In neurological disorders which occur shortly after treatment, also described in the published literature as “stroke”, the presence of intracerebral clots has not been proved. These events do not appear to reflect thromboembolic disease [84–86, 103, 113, 114]. In such cases air bubbles in the arteries of the brain are reported [114–117].

In cases described as stroke after sclerotherapy, we shall distinguish between two forms: those associated with a paradoxical venous thromboembolism, as a rule with delayed onset of symptoms, which have also been described after various other varicose vein treatment methods [118, 119]; and early-onset strokes with a paradoxical air embolism, a characteristic complication of foam sclerotherapy [104, 120].

It should be noted particularly that all patients with stroke resulting from a paradoxical air embolism after sclerotherapy recover completely or almost completely. To date no significant aftereffects have been reported in these cases [120].

Individual cases of confirmed stroke or TIA have been described after both liquid and foam sclerotherapy; they occur after an interval and are associated with paradoxical thromboembolism [103, 117, 121–125].

#### Recommendation 8

In patients who have presented neurological symptoms, including migraine, after previous sclerotherapy, the following should be considered:

- The patient should remain lying down for longer after the injection.
- Injection of large volumes of foam should be avoided, or liquid sclerosant should be used instead.
- The patient should avoid carrying out the Valsalva manoeuvre soon after the injection.
- Decide on a case-by-case basis (considering a risk–benefit analysis based on the indications).

### Deep vein thrombosis (DVT) and lung embolism (LE)

In Table 1, distal DVT is included under “serious complications”, although in individual cases it may be a “benign complication”, e.g. in the case of an asymptomatic calf vein thrombus. There are insufficient published data to assess the real frequency of DVT after liquid sclerotherapy. Most studies on the effectiveness of liquid sclerotherapy are old and were carried out without duplex ultrasound examination. In most studies there is no clear distinction between symptomatic and asymptomatic DVT, although the clinical consequences are usually distinguishable [126].

Severe thromboembolic events (proximal DVT, lung embolism) very seldomly occur after sclerotherapy [127, 128]. The total frequency of thromboembolic events is less than 1%; the frequency of DVT reported in the meta-analyses of Jia and of Dermondy is 0.6% [129, 130]. Deep vein thrombi are mostly distal. Most cases are discovered during routine follow-up examination by duplex ultrasound and are asymptomatic [80, 84, 130]. The injection of large volumes of liquid sclerosant, and more particularly of foam sclerosant, raise the risk of a thrombus [71, 75, 113, 131]. This is equally true of patients with a known history of thromboembolism or thrombophilia [7]. For patients with these risk factors a precise risk–benefit analysis shall be carried out and additional precautions should be taken [75, 77, 132]. Other risk factors, like overweight or insufficient mobility, should also be considered.

#### Recommendation 9

In patients with a high risk of thromboembolism, e.g. with a history of recurrent SVT and/or DVT or known severe thrombophilia, the following should be considered:

- Use of a thrombus prophylactic drug in accordance with the recommendations on thrombus prophylaxis in current guidelines.
- Physical prophylaxis (compression, exercise).
- Avoid injections of large volumes of foam sclerosant.
- Decide on a case-by-case basis (considering a risk–benefit analysis based on the indications).

### Superficial vein thrombosis

Frequencies between 0 and 45.8% are reported in the literature, with a mean of 4.7% [80, 85, 129]. The definition of superficial vein thrombosis after sclerotherapy is controversial in the literature. An inflammatory reaction in the *injected* sector of vein is usually a keloid reaction to sclerotherapy, which—as long as it does not exceed a normal size—should not be interpreted as a superficial vein thrombosis; on the other hand, a superficial vein thrombosis in an *uninjected* vein, or which clearly extends beyond the injected sector, would meet the definition of a superficial vein thrombosis. According to this interpretation, superficial vein thrombosis does occur after sclerotherapy; however, its real frequency is unknown.

### Damage to motor nerves

The incidence of nerve damage after sclerotherapy is very low, lower than with other treatment methods for varicose veins [133].

### Hyperpigmentation

Transient skin pigmentation is reported with a frequency between 0.3 and 30% [93, 134]. In general the pigmentation disappears slowly over a period of weeks or months [135]. The incidence of hyperpigmentation is probably higher after foam sclerotherapy than liquid sclerotherapy [80]. To reduce the frequency of hyperpigmentation, intravascular clots should be removed by needle aspiration or squeezed out through a stab incision [136, 137].

#### Recommendation 10

To reduce the risk of hyperpigmentation, superficial clots can be removed.

### Matting

Matting describes the repeated appearance of fine spider veins in the region of a vein which has already been treated by sclerotherapy or another ablation technique (stripping, laser); it is an unpredictable individual reaction of the patient. Matting [138] can also occur after operative or thermal ablation of a varicose vein [93]. In many cases the cause is non-treatment or insufficient treatment of the underlying reflux. High initial concentrations or large volumes of sclerosant can likewise lead to inflammation or excessive obstruction of the veins, with resulting angiogenesis. Treatment of matting should focus on the possible underlying reflux and the remaining open veins; the best treatment is with low concentrations of sclerosant or stripping [85, 139].

### Other

Other transient general or local reactions after sclerotherapy are tightness in the chest, vasovagal syncope, nausea, metallic taste, intravascular clot, haematoma, ecchymosis at the injection site, pain at the injection site, local swelling, induration, wheals, blistering and erythema. Complications may also be caused by compression bandages, e.g. blistering in the region of a sticking plaster.

#### Recommendation 11

To increase the general safety of foam sclerotherapy the following should be considered:

- Injection of very viscous foam in the varicose veins (C2).
- The patient should not move, particularly his/her leg, for several minutes after the injection; the patient should not carry out the Valsalva manoeuvre.

The type of gas that should be used to generate the foam (air or “physiological” gas) is still in dispute. If large volumes of foam are injected, a foam sclerosant with a low nitrogen content appears to reduce the early, reversible side effects [140, 141]. In patients treated with small amounts of CO_2_/O_2_ foam or air foam, no advantages could be demonstrated for the CO_2_/O_2_ foam in terms of causation of neurological disorders [142, 143].

## 6 Patient information

### Recommendation 12

Before sclerotherapy patients shall be informed of the following:

- Alternative treatment methods with their advantages and disadvantages.
- Details of the sclerotherapy procedure and post-operative treatment.
- Serious risks and complications.
- Frequent side effects.
- Explanation of rare and minor side effects in non-medically indicated sclerotherapy.

### Recommendation 13

With respect to the expected outcome of sclerotherapy, patients should be informed of the following:

- Short- and medium-term controls may be necessary.
- Repeat treatment may be needed in some cases, especially in treatment of large varicose veins.
- Foam sclerotherapy is more effective than liquid sclerotherapy for subcutaneous varicose veins.
- Ultrasound-controlled foam sclerotherapy can avoid the need for an intra-arterial injection.
- Certain side effects may be more frequent with foam (see Complications and risks section).

## 7 Diagnosis before sclerotherapy and documentation

Successful sclerotherapy requires a methodical procedure. Treatment is usually applied in sequence from proximal to distal reflux sources, and from larger to smaller varicose veins. A comprehensive diagnosis shall therefore be carried out before treatment [59].

Standard diagnosis of patients with chronic vein disease includes the patient’s medical history, and clinical and duplex ultrasound examination by a trained doctor. In cases of spider veins and reticular varices, examination with uni- or bidirectional Doppler ultrasound instead of duplex ultrasound may be sufficient. However, the general trend is towards duplex ultrasound for the initial examination also in these cases.

Duplex ultrasound examination is carried out with the patient standing, and is particularly good for identifying incompetent saphenous veins, subcutaneous veins (tributaries) and connections to the deep vein system, for clarifying post-thrombotic alterations, and for planning treatment [144–147]. Duplex ultrasound should also always be used to show incompetent terminal and/or pre-terminal valves. Duplex ultrasound offers substantial advantages over Doppler ultrasound for pre-therapeutic evaluation of saphenous vein incompetence, including measuring vein diameters [148].

### Recommendation 14

Before sclerotherapy, a diagnosis shall be obtained, including medical history and clinical and duplex ultrasound examinations. In cases of spider veins and reticular varices, examination with uni- or bidirectional Doppler ultrasound instead of duplex ultrasound can be sufficient.

Patients with new and/or recurrent varicose veins after previous treatment are recommended to have duplex ultrasound before sclerotherapy [149, 150]. In cases of vessel malformation, thorough duplex ultrasound is also recommended. In some cases further examinations are necessary to clarify the anatomical and haemodynamic situation [51, 151, 152].

Functional examinations (e.g. photoplethysmography, phlebodynamometry, venous occlusion plethysmography) should also be considered. Other imaging techniques (e.g. phlebography) should only be used in exceptional cases [62, 153, 154].

### Recommendation 15

Patients with recurrent varicose veins and patients with vessel malformations shall have a duplex ultrasound examination before sclerotherapy.

It is not necessary to examine specifically for the presence of a right-to-left shunt or thrombophilia before foam sclerotherapy [75].

### Recommendation 16

Routine examination for a right-to-left shunt or the presence of thrombophilia factors in a clot system can be omitted.

The type of treatment, the number of treatments (injections and sessions), the medicinal products injected, volumes, concentrations and the proportions of the foam ingredients should be documented, including details of the veins treated (mapping).

## 8 Sclerotherapy of varicose veins

### Polidocanol (Lauromacrogol 400)

A variety of different sclerosants have been used to treat varicose veins in recent decades, depending on national policies and traditions. In Germany, the only product authorised for use in the sclerotherapy of varicose veins is Aethoxysklerol® (Chemische Fabrik, Kreussler & Co. GmbH, Wiesbaden, Germany) [155], with the active ingredient polidocanol (Lauromacrogol 400).

Polidocanol is available in the following concentrations: 0.25%, 0.5%, 1%, 2% and 3% (corresponding to 5 mg, 10 mg, 20 mg, 40 mg and 60 mg in a 2 ml ampule).

Polidocanol is a non-ionic detergent and a local anaesthetic. A dose of 2 mg polidocanol per kilogram bodyweight per day should not be exceeded (see German product information for Aethoxysklerol® [155]). Thus, for a patient with a bodyweight of 70 kg, a maximum of 140 mg of polidocanol can be injected for varicose vein sclerotherapy—regardless of the amount recommended for medical purposes.

140 mg polidocanol are contained in:

- Aethoxysklerol® 0.25%: 56 ml injection solution.
- Aethoxysklerol® 0.5%: 28 ml injection solution.
- Aethoxysklerol® 1%: 14 ml injection solution.
- Aethoxysklerol® 2%: 7 ml injection solution.
- Aethoxysklerol® 3%: 4.6 ml injection solution.

Sclerotherapy can be carried out with or without ultrasound control and with liquid or foam sclerosant.

### 8.1 Liquid sclerotherapy

#### Recommendation 17

The following recommendations on concentrations and amounts per injection for liquid sclerotherapy should be observed. Concentrations and amounts are reference values and can be adapted according to the therapist’s assessment (Tables 2 and 3).

**Table 2:** Recommended amounts per injection for polidocanol in *liquid* sclerotherapy with single injections [155]

Indications	Volume/injection point
Spider veins (C1)	Up to 0.2 ml
Reticular varices (C1)	Up to 0.5 ml
Varicose veins (C2)	Up to 2.0 ml

**Table 3:** Recommended concentrations in *liquid *sclerotherapy with polidocanol [155]

Indications	Concentration (%)
Spider veins	0.25–1.0
Reticular varices	0.5–1
Small varicose veins	1
Medium-sized varicose veins	2–3
Large varicose veins	3

#### 8.1.1 Spider veins and reticular varices (C1)

##### Recommendation 18

The following recommendations should be observed for liquid ablation of spider veins and reticular varices (C1):

- Puncture and injection of spider veins and reticular varices are carried out with the limb in the horizontal position.
- A low-friction syringe is recommended.
- A small cannula (up to 32 G) can be used.
- An air-block system can be used.
- The outcome may be improved by repeated sessions.
- In spider veins and reticular varices, discoloration of the vein immediately after the start of injection shows that the sclerosant is forcing out the blood and that the injection is intravasal.
- If the skin round the injection point turns white during injection, the injection shall be stopped immediately to avoid skin damage.
- In liquid ablation, as a rule, the sclerosant is slowly injected intravenously, if possible in a fractionated dose and controlling the intravasal position of the cannula.
- Severe pain during injection may indicate paravasal or even intra-arterial injection. In this case injection shall be stopped immediately.
- Diaphanoscopy can be used to detect invisible tributary or perforator veins.

#### 8.1.2 Varicose veins (C2)

##### Recommendation 19

The following recommendations should be observed for liquid ablation of varicose veins (C2):

- The vein can be punctured with a free needle (“open needle”) or a cannula attached to the syringe (“closed needle”).
- Avoid puncturing a perforator vein or saphenofemoral junction directly.
- Low-friction syringes and cannulae of different diameters are recommended according to the indication.
- Injection systems: the injection can be carried out:
  - with a cannula attached to the syringe (“closed needle”); the syringe is filled with sclerosant (e.g. 2.5–5 ml),
  - with a butterfly catheter as an option for varicose veins lying immediately under the skin (preferably with a short silicone tube due to the stability of the foam),
  - with a short catheter (e.g. Braunüle® [B. Braun Melsungen AG, Melsungen, Germany]) as an option for saphenous veins and with the possibility of an injection afterwards,
  - with a long catheter as an option for varicose saphenous veins.
- After puncturing the skin with the cannula, the intravasal position is checked by allowing the blood to flow back or by aspiration, as appropriate.
- Several injections can be applied per session along the treated vein.
- Injection should be carried out with patient lying down.
- As a rule, the sclerosant is slowly injected intravenously, if possible in a fractionated dose and controlling the intravasal position of the cannula or short catheter.
- Severe pain during injection may indicate paravasal or even intra-arterial injection. In this case injection shall be stopped immediately.

### 8.2 Foam sclerotherapy

Sclerotherapy with foam sclerosants has been reported in the literature for many years [156]. Since foam was formally authorised in 2009, foam sclerotherapy has been practised with improving techniques, especially for the treatment of large-diameter veins [8, 128, 155, 157].

Detergent-type sclerosants, like polidocanol, can be converted into a fine-bubble foam by special techniques. In Tessari’s method the foam is produced by turbulent mixing of liquid and air in two syringes, connected by a three-way stopcock. In Tessari’s original technique, the proportion of sclerosant to air was 1 + 4 [156, 158]. In the double-syringe system (DSS), polidocanol sclerosant is mixed with air in the proportion of 1 + 4 by turbulent mixing in two syringes connected by a special two-way connector. At low concentrations of sclerosant, the resulting foam is relatively unstable; at higher concentrations it becomes more stable and viscous. There are no reports of side effects other than those attributable to the use of unsterile air for foam production [159].

Foam sclerotherapy can be performed with or without ultrasound control. Easily visible or palpable varicose veins can be treated simply, without ultrasound control [160, 161].

#### Foam production

##### Recommendation 20

For all indications, a three-way stopcock (Tessari method) or a two-way connector (DSS method)—or a similarly appropriate method—should be used for production of the sclerotherapy foam.

##### Recommendation 21

For all indications, ambient air or a mixture of carbon dioxide and oxygen should be used for the gas component in foam production.

##### Recommendation 22

A mixture of liquid sclerosant and gas in proportions of 1 + 4 (one part liquid to four parts gas) or 1 + 5 should be used for sclerotherapy foam production. For treatment of large-calibre varicose veins (C2), a homogeneous, viscous, fine-bubble foam shall be used. The proportion of liquid can be increased, especially in the case of low-concentration sclerosant.

##### Recommendation 23

The interval between foam production and injection should be as short as possible.

##### Recommendation 24

In foam sclerotherapy of large veins the cannula should be no smaller than 25 G; in so far as possible, low-silicon materials should be used and if a silicon tube is used (with a butterfly), it should be as short as possible, otherwise the foam quality will be affected.

Any alteration in the physical properties (e.g. cooling or heating) can alter the safety profile of the sclerosant used.

#### Foam volumes

There is no evidence-based specification for the maximum volume of foam per session. In the previous European consensus on foam sclerotherapy, the opinion of experts was that a volume of 10 ml of foam should be regarded as the safe maximum [75]. The incidence of thromboembolic complications and temporary side effects (e.g. vision disorders) rises with larger volumes of foam [115, 131].

##### Recommendation 25

In routine cases a maximum volume of 10 ml of foam per day/session should not be exceeded. Larger volumes of foam may however be used after carrying out an individual risk–benefit analysis.

#### Concentration of the sclerosant for foam sclerotherapy

##### Recommendation 26

The following concentrations should be observed in proportion to the diameter of the treated vein segment. The suggested concentrations and amounts are reference values and may be adapted according to the therapist’s assessment (Table 4).

**Table 4:** Recommended concentrations of polidocanol for *foam *sclerotherapy [5, 8, 11, 19, 21, 23, 25–29, 31, 43–46, 51–53, 58, 60, 75, 79, 162–164]

Indications		Polidocanol concentration (%)
Spider veins		Up to 0.5
Reticular varices		Up to 1
Varicose tributary veins		Up to 2
GSV, SSV	<4 mm	1
	≥4 to ≤8 mm	1–3
	>8 mm	3
Incompetent perforator veins		1–3
Recurrent varicose veins		1–3
Venous malformations		1–3

In most studies with incompetent perforator veins, recurrent varicose veins and venous malformations, 1% polidocanol is applied [12, 56].

### 8.3 Ultrasound-guided sclerotherapy

Ultrasound-guided sclerotherapy with liquid and foam sclerosants has proved to be a useful complement to the various treatments available for varicose veins. In particular it is suitable for treatment of varicose saphenous veins (GSV and SSV), tributaries and perforator veins, and in cases of recurrent varicose veins and venous malformations [21, 30, 54–57, 165–167].

#### Recommendation 27

The following recommendations should be observed for ultrasound-controlled sclerotherapy:

- The vein segment to be treated and the nearby arteries are examined in ultrasound before the puncture is made.
- When treating incompetent saphenofemoral junctions and varicose saphenous veins it is recommended that the vein should be punctured in the proximal thigh region (great saphenous vein and anterior accessory saphenous vein) or the proximal calf (small saphenous vein).
- In all other cases the vein should be punctured at the safest and most accessible point.
- The vein should be shown in ultrasound lengthwise and/or in cross section.
- The vein is punctured under ultrasound control and the point of the cannula is placed in the centre of the vessel lumen.
- Backflow of blood into the cannula or catheter is checked and a few drops of liquid sclerosant or bubbles of foam are injected into the vein and controlled on the ultrasound screen before the actual injection.
- The injection is performed under ultrasound control.
- Foam is more suitable than liquid for ultrasound-controlled sclerotherapy because the bubbles contrast with the echo-poor vessels, allowing the sclerosant to be seen.
- After injection ultrasound is used to control the distribution of the sclerosant and the reaction of the vein (including venospasm).

### 8.4 Mechanochemical endovenous ablation (MOCA)

Mechanochemical endovenous ablation is a combination of mechanical damage to the vein wall and a chemical sclerotherapy reaction. A wire is introduced through a catheter into the saphenous vein and pushed up to the junction; during injection, usually of liquid sclerosant, the point is rotated rapidly. The combination of mechanical damage to the endothelium of the saphenous vein and the effect of the sclerosant is supposed to result in a better vein occlusion rate [168, 169]. The maximum daily doses for sclerosant injection shall be observed.

In several case series and non-randomised studies, high initial occlusion rates and little pain were reported [170–175]. MOCA was compared with radiofrequency ablation (RFA) in a prospective randomised study [176]. This showed that the pain was significantly less than after RFA, and occlusion rates, improvement in clinical findings and quality of life were comparable after 2 years. No longer-term outcomes are available. The side effects profile is similar to that of other sclerotherapy procedures.

#### Recommendation 28

Mechanochemical endovenous ablation can be used as an alternative to the other sclerotherapy methods for saphenous vein sclerotherapy.

## 9 Post-operative treatment after sclerotherapy

### Recommendation 29

The following aspects of post-operative treatment after sclerotherapy should be considered:

- Watch carefully for any signs of undesired reactions.
- In addition to sclerotherapy, the treated limb can be treated with compression, either with a compression stocking or compression bandaging.
- The outcome of sclerotherapy of spider veins can be improved by daily wearing of compression stockings (23–32 mm Hg) for up to 3 weeks after treatment [187].
- Longer-term immobility after sclerotherapy can increase the risk of thromboembolic events.
- Remaining clots can be punctured where possible (with or without ultrasound control) in the post-operative check-up.

Walking a long distance after sclerotherapy is widely recommended; however, there are no indications in the literature to date either for or against this measure.

## 10 Outcome control after sclerotherapy

Assessment of the effectiveness of sclerotherapy comprises clinical, morphological and haemodynamic aspects.

For spider veins and reticular varices, a clinical check-up is sufficient.

Clinical outcome:

- Clinical assessment in routine practice: presence/absence or improvement of varicose veins in the treated area, assessed by the doctor and/or patients.
- The presence of venous ulcer, oedema, haemorrhage, inflammation, etc. belong to the clinical outcome.
- Symptom reporting: if necessary (e.g. in the context of scientific research), differentiated and standardised symptom scores like the Venous Clinical Severity Score (VCSS) and Patient-Reported Outcome Scores can be used.

Morphological and haemodynamic outcome:

The morphology of the treated vein can be assessed with duplex ultrasound by the compressibility with the patient standing up. The appropriate settings shall be used for duplex ultrasound [146]. The patency, occlusion (total or partial) or disappearance of the vein shall be checked. The examination should also include dynamic manoeuvres as per the UIP consensus [147].

See Table 5 for the findings that can be determined by duplex ultrasound.

**Table 5:** Findings in post-therapeutic control by duplex ultrasound

Circulation and reflux	Morphology and haemodynamics	
No circulation	*Patency/occlusion*	Complete disappearance of the treated vein
Antegrade circulation without reflux (<0.5 s)		Total occlusion (no compressibility) of the treated vein segment
Reflux <1 s		Partial occlusion of the treated vein segment
Reflux >1 s		Total patency of the treated vein segment
	*Vein measurements*	Diameter before treatment
		Internal diameter after treatment
		Length of occluded segment
		Length of patent segment

These examination parameters can be used for all endovenous treatment procedures (laser, radiofrequency, sclerotherapy) and should make comparison easier, especially in scientific studies.

From a clinical point of view, regression of the varicose veins and/or venous symptoms is regarded as therapeutic success.

The disappearance or total occlusion of the treated vein in duplex ultrasound is regarded as the optimum therapeutic outcome.

Clinical improvement with occlusion of the treated vein, but with short open sections containing occasional circulation, can be judged a therapeutic success at least in the short to medium term.

After sclerotherapy the findings in duplex ultrasound can present a wide spectrum of outcomes, which will not necessarily agree with the clinical outcome.

In some cases improved vein function can be shown by pre- and post-treatment functional examinations (e.g. plethysmography, vein pressure measurements) [62, 153, 154].

### Recommendation 30

With spider veins and reticular varices (C1), the success of treatment can be assessed in the check-up after sclerotherapy from the clinical outcome. With varicose veins (C2) and venous malformations, both clinical and ultrasound examinations should be carried out.

## 11 Effectiveness

Sclerotherapy with liquid and foam sclerosants is a safe and effective procedure for treating spider veins, reticular varices and subcutaneous varicose veins [5, 8, 9, 20, 25, 29, 34, 59, 60, 73, 164, 167].

Sclerotherapy with liquid polidocanol is the method of choice for treating spider veins and reticular varices, resulting in an improvement of more than 90% after treatment [20, 25–29, 58]. Foam sclerotherapy is an alternative procedure for the ablation of spider veins and reticular varices, with similar occlusion rates and side effects, as long as low concentrations are used in a rather liquid foam [8, 29].

Foam sclerotherapy is significantly more effective than liquid sclerotherapy for varicose saphenous veins [5, 7–9, 27]. The occlusion rate depends on the vein diameter, the concentration of the sclerosant and the volume of foam injected [19, 27]. Compared with endovenous thermal ablation and stripping operations, foam sclerotherapy has a higher medium-term rate of re-channelling [11, 12, 65, 67, 68]. The improvements in quality of life and symptoms are similar [11, 14–16, 65]; however, the improvement in quality of life achieved after 5 years is superior to that of EVLA and stripping operations [18].

There is no firm evidence for an improvement in occlusion rate or a reduction of side effects by keeping the limb raised, compression of the junction with the ultrasound probe or use of tumescent solution to reduce vessel diameter [101, 177, 178].

Foam sclerotherapy of incompetent saphenous veins with a long catheter is also effective [13, 47, 177, 179–184].

Follow-up sclerotherapy of partially re-channelled segments of vein is recommended and improves the medium-term outcome [185, 186].

Sclerotherapy of veins in the region of a venous ulcer improves the healing rate [43–50]*.* Early ablation of the incompetent saphenous vein together with peri-ulcer sclerotherapy has proved effective in the treatment of venous leg ulcers. Foam sclerotherapy accelerates healing, comparable with endovenous thermal procedures [74].

Foam sclerotherapy is more effective than liquid sclerotherapy for treating venous malformations [51–53].

Foam sclerotherapy is also effective for treating new and recurrent varicose veins after previous treatment, varicose saphenous tributaries, other superficial varicose veins and incompetent perforator veins [19, 21, 23, 31–35, 39].

Compression treatment with medical compression stockings or bandages improves the outcome of sclerotherapy of spider veins [187–190] and may reduce the frequency of pigmentation [188–190]. There is still little evidence for the effectiveness of compression after sclerotherapy of saphenous veins [191, 192]. In a study in which compression stockings of three different compression classes were worn for 3 weeks after sclerotherapy, the higher the compression pressure, the lower the need for follow-up sclerotherapy [193]. Even selective positive eccentric compression can reduce the recurrence rate [194]. Local eccentric compression increases the local pressure in the sclerotherapy region significantly and may improve the effectiveness of sclerotherapy [195]. Treatment with topical corticosteroids immediately after sclerotherapy, on the other hand, apparently offers no benefits in terms of the appearance of inflammatory side effects [196].
